# Ensuring safety and security for Ecuador's rural health doctors: a call to action

**DOI:** 10.3389/fpubh.2025.1607070

**Published:** 2025-05-14

**Authors:** Esteban Ortiz-Prado, Luis Fernando Merlo-Chaves, Jorge Vasconez-Gonzalez, Isaac A. Suarez-Sangucho, Juan S. Izquierdo-Condoy

**Affiliations:** ^1^One Health Research Group, Universidad de Las Américas, Quito, Ecuador; ^2^Faculty of Medicine at the Universidad de las Américas, Quito, Ecuador

**Keywords:** rural health doctors, safety, security, insecurity, Ecuador

As part of physicians' professional training, many countries have implemented a mandatory year of social service (also known as community or rural medicine) to enhance student experience and help reduce healthcare disparities in rural areas. Some programs, such as the one introduced by Mexican legislature, have integrated this into their medical undergraduate curricula (1). In other countries, like Ecuador, social service is a requirement for newly graduated health professionals. In its current design, Ecuador's rural medicine year requires physicians to provide primary care focused on health promotion and prevention to residents in often remote and hard-to-reach communities (2, 3).

Since its inception in the 1970s, rural medicine has become a fundamental axis of the Ecuadorian National Health System. However, despite significant changes in the country over the decades, the program has remained largely stagnant. The first official regulations for the program were published in 1991, and there is little evidence that its outcomes have been thoroughly evaluated since (4). In 2012, the Ministry of Health introduced the Manual of the Comprehensive Family, Community, and Intercultural Health Care Model (MAIS–FCI) and established Primary Health Care Centers as gateways to the health system. These centers are intended to solve 80% of the population's health problems, particularly in rural areas (5, 6). They are the primary posts for healthcare professionals completing their rural service, with staffing based on geographic location, access, poverty levels, epidemiological profiles, and malnutrition rates, among other factors (7).

Despite their importance, rural doctors in Ecuador face numerous daily challenges, including a lack of medications, poor infrastructure, insufficient medical equipment, and delays in salary payments (8). Unofficial reports of limited resources and precarious working conditions for these professionals are common. For example, in 2021, a rural doctor sustained a head injury while performing community activities; in 2023, a rural doctor died in a plane crash while in route to a remote community; and in 2024, five rural doctors were killed in a car accident while traveling for their rural service (9–12). Additionally, the Guayas Provincial Medical College has received over 60 reports of extortion attempts against doctors since May 2023, reflecting the significant risks faced by rural healthcare workers, exacerbated by rising crime rates in Ecuador (13–16).

On January 1, 2024, 5,670 health professionals began their year of rural service. However, by May 2024, Dr. Steven Aguirre, a young physician working in “El Empalme” in Ecuador's coastal region, was murdered by criminals who attempted to kidnap him while traveling to work. Dr. Aguirre had already been the target of a kidnapping attempt in 2023 and was temporarily transferred to another medical unit for 45 days, only to be sent back to the same dangerous location (17). This tragedy, as many others, has filled the medical community with deep pain and indignation as it highlights the risks that young physicians are submitted to in lieu of fulfilling this requirement. Dr. Aguirre's death, directly linked to the growing violence and extortion against rural doctors, demands immediate and decisive action. His murder, particularly after he bravely reported previous extortion attempts, exposes the severe and excessive risks faced by those providing healthcare to Ecuador's most vulnerable populations.

The Federation of Rural Health Professionals reported receiving between 600 and 700 complaints from rural health professionals in 2024 alone, related to theft, threats, intimidation, and harassment, particularly in coastal and Amazonian provinces (18). While past security threats mainly involved opportunistic criminals, the current situation in rural Ecuador is far more alarming, as organized crime has taken hold of vulnerable regions, with competing gangs fighting for control. Physicians are extorted for payments ranging from $100 to $400 a week, depending on which gang controls the area. Armed men have even entered health centers demanding money in exchange for “protection” (15, 16). Despite repeated complaints to authorities, little has been done to safeguard these professionals, forcing them to pay extortion fees from their modest salaries for their safety. Since the rural service lasts only 1 year, extortionists are continually supplied with new victims, who often prefer silence to risking their own or their family's safety. This dynamic may be contributing to the exodus of young doctors seeking safer opportunities abroad, further worsening the healthcare crisis in Ecuador's rural areas.

The current healthcare system is failing its human contingent. Despite repeated requests for help, Ecuadorian authorities throughout different administrations have ignored the escalating dangers of the current system in favor of keeping medical centers staffed. The absence of protocols to protect rural doctors, even after numerous incidents like Dr. Aguirre's initial report, is unacceptable. Immediate steps must be taken to address this crisis. Some proposed actions include:

1. **Increased security:** deploy a visible and effective security presence in rural health centers. Given that healthcare is a high-priority strategic area, law enforcement should be stationed to ensure the continued provision of healthcare to all populations. Work schedules and shifts should be adjusted in high-risk areas and police escorts should be provided to healthcare workers traveling to and from their workplaces.
2. **End impunity:** conduct a swift and thorough investigation into Dr. Aguirre's murder and bring those responsible to justice. As investigations have uncovered networks of corruption involving high-ranking public officials, it is crucial to connect these activities to the crime surrounding health centers and hospitals. Given that healthcare is one of the main objectives of the National Development Plan (Strategic Axis 1) (19), legislative bodies should consider classifying organized criminal acts targeting healthcare workers as acts of terrorism. It must be made clear that violence against healthcare professionals will not be tolerated.
3. **Reevaluate mandatory rural year:** while recognizing the importance of providing healthcare to rural communities, alternative solutions that prioritize the safety and wellbeing of healthcare professionals must be explored. Strategies such as short, mandatory rotations for postgraduate students in rural hospitals, stimulating development of medical specialties programs based in hospitals outside capital cities and ensuring economic conditions to prevent migration of trained professionals to main cities could help alleviate the workforce gap in these populations.

We hope that this report, along with the tragic death of Dr. Aguirre, will prompt authorities to take depoliticized actions aimed at protecting the rights, wellbeing, and safety of healthcare professionals delivering rural health services in Ecuador.
